# Radiative Cooling for Energy Sustainability: From Fundamentals to Fabrication Methods Toward Commercialization

**DOI:** 10.1002/advs.202305067

**Published:** 2023-11-10

**Authors:** Sunae So, Jooyeong Yun, Byoungsu Ko, Dasol Lee, Minkyung Kim, Jaebum Noh, Cherry Park, Junkyeong Park, Junsuk Rho

**Affiliations:** ^1^ Graduate School of Artificial Intelligence Pohang University of Science and Technology (POSTECH) Pohang 37673 Republic of Korea; ^2^ Department of Mechanical Engineering Pohang University of Science and Technology (POSTECH) Pohang 37673 Republic of Korea; ^3^ Department of Electro‐Mechanical Systems Engineering Korea University Sejong 30019 Republic of Korea; ^4^ Department of Biomedical Engineering Yonsei University Wonju 26493 Republic of Korea; ^5^ School of Mechanical Engineering Gwangju Institute of Science and Technology (GIST) Gwangju 61005 Republic of Korea; ^6^ Department of Chemical Engineering Pohang University of Science and Technology (POSTECH) Pohang 37673 Republic of Korea; ^7^ POSCO‐POSTECH‐RIST Convergence Research Center for Flat Optics and Metaphotonics Pohang 37673 Republic of Korea

**Keywords:** metamaterials, metasurfaces, nanostructures, radiative cooling, sustainable energy

## Abstract

Radiative cooling, a technology that lowers the temperature of terrestrial objects by dissipating heat into outer space, presents a promising ecologically‐benign solution for sustainable cooling. Recent years witness substantial progress in radiative cooling technologies, bringing them closer to commercialization. This comprehensive review provides a structured overview of radiative cooling technologies, encompassing essential principles, fabrication techniques, and practical applications, with the goal of guiding researchers toward successful commercialization. The review begins by introducing the fundamentals of radiative cooling and the associated design strategies to achieve it. Then, various fabrication methods utilized for the realization of radiative cooling devices are thoroughly discussed. This discussion includes detailed assessments of scalability, fabrication costs, and performance considerations, encompassing both structural designs and fabrication techniques. Building upon these insights, potential fabrication approaches suitable for practical applications and commercialization are proposed. Further, the recent efforts made toward the practical applications of radiative cooling technology, including its visual appearance, switching capability, and compatibility are examined. By encompassing a broad range of topics, from fundamental principles to fabrication and applications, this review aims to bridge the gap between theoretical research and real‐world implementation, fostering the advancement and widespread adoption of radiative cooling technology.

## Graphical abstract

Radiative cooling technologies garner significant interest as ecologically‐benign solutions for future energy sustainability. This comprehensive review offers a well‐organized exploration of radiative cooling, encompassing its foundational design principles, fabrication techniques, potential methods for practical implementation, and real‐world applications. Its overarching aim is to guide researchers seeking successful commercialization within this field.

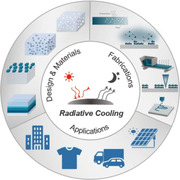

## 1 Introduction

Cooling technologies have been important since ancient times, and many novel technologies have been developed. However, most current cooling systems rely on vapor compression, necessitating the use of refrigerants and external energy; and therefore, raising environmental concerns. It is worth noting that ≈10% of energy consumed in the United States is used for cooling the interiors of buildings.^[^ ^1^ ^]^ Ever‐increasing concerns about global warming, and the imperative to achieve net‐zero carbon emissions are driving research to develop ecologically‐benign, energy‐efficient methods to cool buildings, vehicles, industrial equipment, and the human body.

Passive radiative cooling (RC) may present a future sustainable cooling technology that operates without external energy. By harnessing the coldness of the universe, RC dissipates excessive heat of terrestrial objects to the universe by thermal radiation. This is in sharp contrast to other cooling technologies,^[^ ^2^, ^3^ ^]^ which mostly deposit heat elsewhere on Earth. Coincidentally, the atmosphere on a cloud‐free day is transparent in the main range of wavelengths (8 ≤ *λ* ≤ 13 µm) emitted by objects that have a temperature of ≈300 K. This range is called the atmospheric window (AW). Thus, thermal radiation emitted by these objects can be dissipated into space without being absorbed by the atmosphere.

RC exploits a ubiquitous heat transfer process of thermal radiation.^[^ ^4^ ^]^ In the early stage,^[^ ^5^, ^6^, ^7^, ^8^, ^9^, ^10^, ^11^ ^]^ fundamental studies investigated the emissivity properties,^[^ ^7^, ^8^, ^9^ ^]^ influence of geological location,^[^ ^12^ ^]^ and influence of weather conditions^[^ ^13^ ^]^ on atmospheric transmittance. The research identified several materials, including polymers,^[^ ^5^ ^]^ titanium dioxides,^[^ ^7^ ^]^ silicon nitrides,^[^ ^14^ ^]^ and silicon monoxide^[^ ^8^ ^]^ that have great potential for emitting thermal radiation in the AW. Subsequently, sub‐ambient night‐time RC has been achieved by using these bulk materials.^[^ ^6^, ^7^, ^15^, ^16^ ^]^ However, their use in daytime RC was found to be limited because they absorb a significant amount of sunlight. Therefore, a fundamental challenge is posed for daytime RC, which requires the manipulation of electromagnetic properties to achieve both low absorption in the solar spectrum (0.3 ≤ *λ* ≤ 2.5 µm) and high emittance in the AW.

Approaches that utilized several materials and structural effects facilitated the development of sub‐ambient daytime RC over the last decade.^[^ ^17^, ^18^ ^]^ In these approaches, structural designs had provided essential ways to engineer electromagnetic properties over such a broad wavelength range, allowing for sub‐ambient daytime RC by suppressing solar absorption and emitting thermal radiation in the AW. The accessibility of full day‐and‐night RC offers even vast opportunities for energy‐efficient applications for buildings,^[^ ^19^ ^]^ vehicles,^[^ ^20^ ^]^ solar cells,^[^ ^21^ ^]^ and for personal thermal management.^[^ ^22^ ^]^ The significant impact and potential of passive RC has spurred researchers to address the remaining practical challenges over the last few years, including implementing novel fabrication methods and practical functionalities. Consequently, a number of review papers^[^ ^19^, ^23^, ^24^, ^25^, ^26^, ^27^, ^28^, ^29^, ^30^, ^31^ ^]^ have also actively been published to comprehensively summarize the latest trends in RC technologies and propose future directions, all aimed at driving technological advancements in this field. For example, several review papers^[^ ^19^, ^24^ ^]^ have examined the influences of environmental factors on cooling performances, such as climate, temperature, and atmospheric conditions to explore high‐performing radiative coolers. Materials and structural designs have been thoroughly reviewed^[^ ^25^, ^26^ ^]^ to inspire innovative structural designs or guide suitable choices. Other review papers have focused on aspects of the historical development^[^ ^32^ ^]^ of RC technologies or their integration in specific applications such as buildings,^[^ ^28^, ^29^ ^]^ solar energy harvesting,^[^ ^23^, ^30^ ^]^ and personal thermal management.^[^ ^31^, ^33^ ^]^ Meanwhile, a few have examined various fabrication methods^[^ ^27^, ^33^, ^34^ ^]^ for the realization of radiative coolers, but these are often categorized under specific structures and materials. For example, various fabrication methods for organic materials^[^ ^33^ ^]^ and film structures^[^ ^34^ ^]^ have thoroughly been reviewed. However, it is crucial to offer a holistic overview of the diverse fabrication methods that underpin radiative cooler realization, encompassing a wide range of materials and structures. Such an overview is particularly essential to identify potential approaches for real‐world applications and ensure their practical viability.

In this article, we provide a comprehensive review of RC that covers the entire research landscape, encompassing fundamental concepts, structural designs, device fabrication methods, and emerging strategies and applications for commercialization with the aim of bridging the gap between research and practical implementation. We specifically focus on providing a comprehensive overview of various fabrication methods for RC; while, also exploring potential fabrication methods for practicality and real‐world applications. Through this review, we strive to facilitate a deeper understanding of RC and inspire further advancements in the field. We begin by briefly discussing the fundamentals of RC; then, review several structures and their physical mechanisms. Then, we review various methods to fabricate these structures and introduce potential functionalities and practical applications. We conclude by highlighting the challenges and potential of passive RC.(Figure S1, Supporting Information)

## 2 Materials and Structure Designs

RC occurs by energy exchange among the earth, the sun, and the universe. The terrestrial objects’ temperature is det)ermined by the balance among energy absorbed from the Sun (*P*_sun_), atmospheric radiation (*P*_atm_), the thermal emission of the surface (*P*_rad_), and the other heat losses (*P*_non − rad_). By taking into account all energy exchange, the net cooling power $P_{\mathrm{net}\_\mathrm{cooling}}$ is given by:^[^ ^17^ ^]^

(1) $$P_{\mathrm{net}\_\mathrm{cooling}}=P_{\mathrm{rad}}-P_{\mathrm{atm}}-P_{\mathrm{solar}}-P_{\mathrm{non}\_\mathrm{rad}}$$

Each energy term and ideal radiative coolers are discussed in detail in Note S1, Supporting Information.

RC has been realized and developed by evaluations of various photonic structures and materials. Ideal radiative coolers should not absorb the solar spectrum and should perfectly emit thermal radiation in the AW. To meet these requirements, a wide variety of organic and inorganic materials that exhibit phonon‐polariton resonances in the AW have been used. Natural bulk materials that satisfy these properties were studied in the 1970s and 1980s.^[^ ^5^, ^7^, ^15^, ^16^, ^35^, ^36^ ^]^ Most of them are broadband emitters, and their absorption behavior is determined by their bulk material properties. The optical properties of materials commonly used for RC are summarized in **Table** **1**.

**Table 1:** Optical properties of typically used RC materials. Black: refractive index; red: extinction coefficient. Data obtained from ref. [37, 38].

Solar reflection	Metal			
	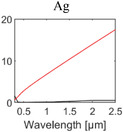		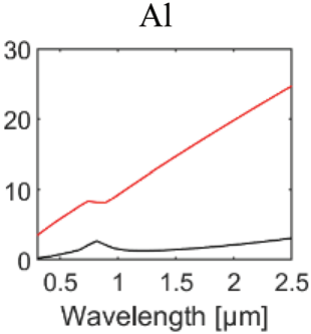	
IR absorption	Inorganic materials			
	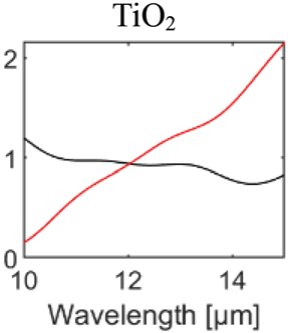	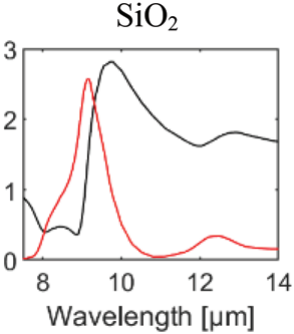	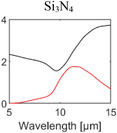	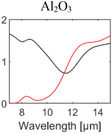
	Polymer			
	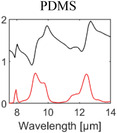		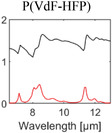	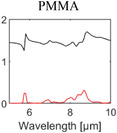

To achieve deep sub‐ambient cooling effect under direct sunlight, the radiative cooler must strongly reflect solar radiation and strongly emit in the AW. These requirements cannot be achieved using only bulk materials as their emission spectrum is fixed. Therefore, to delicately control the electromagnetic properties and thereby increase the RC effect, novel structures must be designed, and their underlying physical phenomena must be exploited.

**Figure 1:**
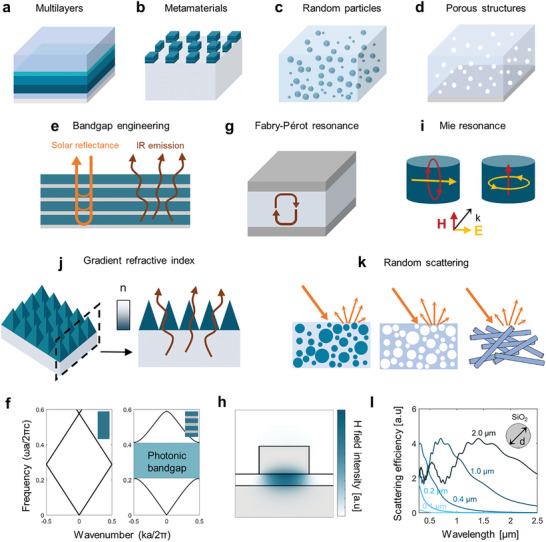
Structural designs for RC and their physical mechanisms. a) Multilayers, b) metamaterials, c) random particles, d) porous structures, e) 1D photonic crystals creating allowed and forbidden modes of light propagation, and f) photonic band diagram of bulk material (left) and 1D photonic crystal (right). Frequencies within the photonic bandgap are forbidden modes and cannot propagate into the structure. g) Resonant absorption generated by cavity modes in metamaterials, h) magnetic field intensity profile of Fabry–Pérot resonance in a metal–insulator–metal structure, i) electric dipole and magnetic dipole resonance in dielectric cylinders, j) effective gradient refractive index produced by the patterned surface, k) light scattering caused by random surface and scatterer geometries, and l) scattering efficiency of silicon dioxide microparticles. Scattering efficiency spectrum differs among particle sizes.

Recent advances in nanophotonics have provided methods to manipulate electromagnetic properties by using tailored nano‐ and micro‐scale structures. Radiative coolers reported to date can be categorized into four structures: multilayer structures, metamaterials, random particles, and porous structures. Multilayer structures refer to photonic designs in which several materials are stacked in layers (**Figure** **1**a). The sequence of materials and layer thicknesses of these structures are engineered to control the spectral responses of radiative coolers. Metamaterials are artificially‐engineered materials composed of subwavelength structures (Figure 1b), which enable delicate manipulation of light–matter interactions, such as triggering electromagnetic resonance and generating an effective gradient refractive index. In contrast to these two structures with precisely defined geometries, random particles (Figure 1c) and porous structures (Figure 1d) are structural designs with random configurations, which exploit a strong optical‐scattering effect. Depending on the target application and environment, different structure and material design strategies are required. In this section, we introduce these four structures, with focus on their physical mechanisms that are exploited to manipulate electromagnetic properties.

### 2.1 Multilayers

A multilayer structure is composed of several materials vertically stacked in layers and can be designed to suppress solar absorption and maximize infrared (IR) emission (Figure 1e). This structure can be understood as a 1D photonic crystal in which two materials that have different refractive indices and thicknesses are periodically stacked. The behavior of wave propagation in the media can be described by the dispersion relation; waves in an isotropic, homogeneous, and dispersionless medium exhibit a linear relation between its frequency and momentum; and thus, show straight curves in the photonic band structure; this result implies that any frequency of light can propagate (Figure 1f, left). On the contrary, in periodic structures, Bragg scattering occurs and a photonic bandgap is formed; that is, when the wavelength of the incident light is comparable to the lattice constant, multiple reflections at each interface create a destructive interference. Consequently, such periodic structures only allow certain frequencies of light to pass through them without scattering while forbidding the propagation of light that has other frequencies. In the photonic band structure, the allowed modes correspond to a set of frequencies on the band (curves), and the forbidden modes correspond to frequencies within the photonic bandgap (Figure 1f, right).

The strategy of using multilayers for RC is to exploit the combination of multiple material properties and utilize the photonic bandgap effect to manipulate electromagnetic properties. Photonic crystals can be used to design a strong reflector or a highly‐selective band‐pass filter for the target wavelength range by stacking a material that has high refractive index alternately with a material that has low refractive index. An aperiodic or quasi‐periodic structure can also be used by optimizing material sequence and layer thicknesses to maximize interference effect and increase broadband reflection in solar spectrum. With well‐chosen material combinations and optimized thicknesses, the bandgap of the structures can be engineered to have the target reflection/emission spectrum that enables high solar reflectivity and strong thermal radiation. Typically, multilayer radiative coolers are composed of many alternating layers and a metal film at the bottom. The alternating layers are responsible for increasing both reflectance in the solar spectrum and thermal emittance in the AW. The metal film provides high reflection from ultraviolet (UV) to near‐infrared (NIR) because of its high conductivity, which cancels out the incident field. Early breakthrough demonstrations of sub‐ambient RC using multilayer structures^[^ ^17^, ^18^ ^]^ have impelled many research communities to widen the range of material candidates and tailor the effectiveness of the devices by tailoring their configurations.^[^ ^39^, ^40^, ^41^, ^42^, ^43^, ^44^, ^45^, ^46^, ^47^, ^48^ ^]^ Owing to the simplicity of multilayer structures, a variety of optimization algorithms can be used to further increase the cooling effect.^[^ ^20^, ^41^, ^49^ ^]^

### 2.2 Metamaterials

Metamaterials are artificially‐engineered materials composed of subwavelength structures. Their subwavelength features allow for delicate manipulation of electromagnetic responses, and thereby, extend the boundaries of material properties far beyond those available in nature.^[^ ^50^ ^]^ Advances in nanofabrication technology have enabled development of photonic designs that use metamaterials to increase absorption within the AW by controlling the light–matter interaction.^[^ ^51^, ^52^, ^53^, ^54^, ^55^, ^56^, ^57^, ^58^ ^]^

Thermal emission in the AW can be increased by using metamaterials to induce electromagnetic resonance. The subwavelength structure geometries and the constituent materials can be designed to exhibit large resonance at target frequencies.^[^ ^50^, ^59^, ^60^ ^]^ Numerous studies have tailored resonant optical cavity modes of metallic and dielectric structures. Metallic resonators support plasmonic resonances induced by surface plasmons, which are coherent and collective electron oscillations confined at dielectric–metal surfaces. The strong photon–electron interactions and extreme light confinement can be increased when combined with cavity modes such as Fabry–Pérot resonance in metal–insulator–metal structures (Figure 1g,h). In contrast, dielectric resonators support multipolar resonant responses called Mie resonances when their characteristic size is comparable to incident light wavelength (Figure 1i). The optical resonances in lossy materials can increase absorption at the corresponding frequency. Therefore, such resonance‐based approaches have been exploited in radiative coolers in various plasmonic^[^ ^51^, ^53^, ^58^ ^]^ and dielectric^[^ ^52^, ^61^ ^]^ systems. In particular, efficient RC requires effective thermal emission through the AW; so, the broadband absorption must cover the entire range of 8 ≤ *λ* ≤ 13 µm. To this end, the structure geometries can be optimized to exhibit multiple resonance,^[^ ^52^, ^53^ ^]^ waveguide modes,^[^ ^51^, ^58^ ^]^ or exploit high loss materials.^[^ ^61^ ^]^

Emission in the AW can also be increased by using micro‐patterned surfaces to generate a gradient refractive index (Figure 1j). Bio‐inspired pyramid structures,^[^ ^54^, ^57^ ^]^ photonic crystals,^[^ ^62^ ^]^ or grating patterns^[^ ^63^, ^64^ ^]^ have been introduced to create an effective index gradient and improve the impedance‐matching condition. The patterned structures can reduce the interface reflection in the mid‐infrared (MIR); and thus, increase the absorption in the AW.

Radiative coolers that use multilayer structures or metamaterials can be tailored to have strong solar reflection and highly selective emission in the IR range. However, they require intricate fabrication steps such as high‐precision lithography or deposition techniques, which limit large‐scale applications of these devices. For practical use of radiative coolers, simpler methods than these are required.

### 2.3 Random Particles

When light traveling in air encounters a particle or a certain object, it interacts with the atoms or molecules of that object. Consequently, the light gets deflected or scattered in different directions. This phenomenon called optical scattering is influenced by various factors such as differences in refractive index, irregular surface, or the size of the particle. By randomly distributing nano/micro‐particles with certain degree of thickness, strong scattering effect is induced which can collectively yield high reflection. In RC technology, particles with high refractive index in the solar spectrum are particularly desirable because optical scattering is driven by the sudden impedance mismatch between the structure and the air (Figure 1k, left). By selecting suitable materials and dispersing them in a random configuration to increase both solar reflection and IR emission, effective RC can be realized. Many approaches aimed to maximize the scattering efficiencies of particles by exploiting the influences of size and material parameters (Figure 1l).

Radiative coolers using random particles have been in the limelight for being compatible with scalable, low‐cost, and reliable production. Various inorganic materials have been evaluated for these particles, mostly in paint‐format, that have high particle concentrations.^[^ ^7^, ^65^, ^66^, ^67^, ^68^, ^69^, ^70^, ^71^, ^72^, ^73^, ^74^ ^]^ Typically, these structures require binding materials to physically bind the particles together and for good mechanical properties. TiO_2_ has a favorable scattering efficiency because it has a high refractive index even compared to typical polymer binders; and therefore, was used in early approaches.^[^ ^7^, ^65^, ^67^, ^68^ ^]^ However, due to its low energy bandgap of 3.0 eV at *λ* < 0.4 µm, it absorbs UV and violet light which together account for 7% of the solar energy;^[^ ^66^ ^]^ and therefore, has limited cooling effect. To mitigate this problem, other UV non‐absorbing materials such as SiO_2_,^[^ ^70^, ^71^, ^72^, ^75^ ^]^ CaCO_3_,^[^ ^73^ ^]^ BaSO_4_,^[^ ^66^, ^74^ ^]^ and Al_2_O_3_^[^ ^72^, ^76^ ^]^ have been exploited.

Paint‐format radiative coolers possessing strong sunlight scattering ability can be used in metal‐free substrates enabling broad range of applications. Hence, strategies to increase the solar reflection of paint‐format coolers have been a major research area. Numerical investigations have sought to optimize the size parameters and size distribution of particles to maximize solar reflection.^[^ ^68^, ^77^, ^78^ ^]^ To analyze different particle coating designs with large thickness, various simulation tools such as Monte Carlo simulation,^[^ ^79^, ^80^, ^81^ ^]^ Lattice Boltzman model,^[^ ^82^, ^83^ ^]^ finite element method,^[^ ^77^ ^]^ and finite‐difference‐time‐domain^[^ ^84^ ^]^ have been used. Other approaches include using multiple materials such as mixing different particles^[^ ^41^, ^72^ ^]^ or utilizing core–shell structures^[^ ^81^, ^85^ ^]^ for complete back‐scattering.

### 2.4 Porous Structures

Porous structures, in principle, use the same strategy as random particles of enhancing optical scattering because the pores can be regarded as the inverse of particles. Randomly distributed air voids generate impedance mismatch between the host medium and pores and can effectively scatter the incoming sunlight (Figure 1k, middle). Porous structures have primarily been implemented in polymers and polymer composites because the fabrication methods are simple, inexpensive, and scalable.^[^ ^86^, ^87^, ^88^, ^89^, ^90^, ^91^, ^92^, ^93^ ^]^ Owing to their molecular vibration modes, polymers generally feature low absorption in the solar spectrum and high absorption in the IR.^[^ ^94^ ^]^ Optimizing the pore size to maximize optical scattering has been a major research goal^[^ ^38^, ^95^ ^]^ as for radiative coolers that use random particles. The scattering‐efficiency spectra of pores depends on their size. Therefore, pores must be fabricated to have hierarchical sizes, from ≈10 nm to ≈10 µm; so that, differently‐sized pores can scatter different wavelength bands to achieve broadband solar reflection from UV to NIR. To produce such polymer structure with broad pore size distribution, a variety of fabrication methods has been proposed and developed such as phase inversion methods using different volatilization rates of solvents, which will be presented in detail in Section 3.

Many reports used networks of porous nanofibers as radiative coolers^[^ ^96^, ^97^, ^98^, ^99^, ^100^, ^101^, ^102^ ^]^ (Figure 1k, right). The fibers were typically produced using electrospinning, which processed materials such as polymers and silk and could achieve high‐throughput fabrication. These structures have enormous potential in applications such as cooling textiles.

With the advances in fabrication technology and material investigations, the diversity of prospective applications of RC technology is continuously expanding. The optimal configuration of a radiative cooling system, including the substrate materials, indoor or outdoor environment, and system enclosures, may significantly vary depending on the specific target application. Depending on these conditions, appropriate design and materials of radiative cooler should be carefully chosen.

## 3 Fabrication Method

**Figure 2:**
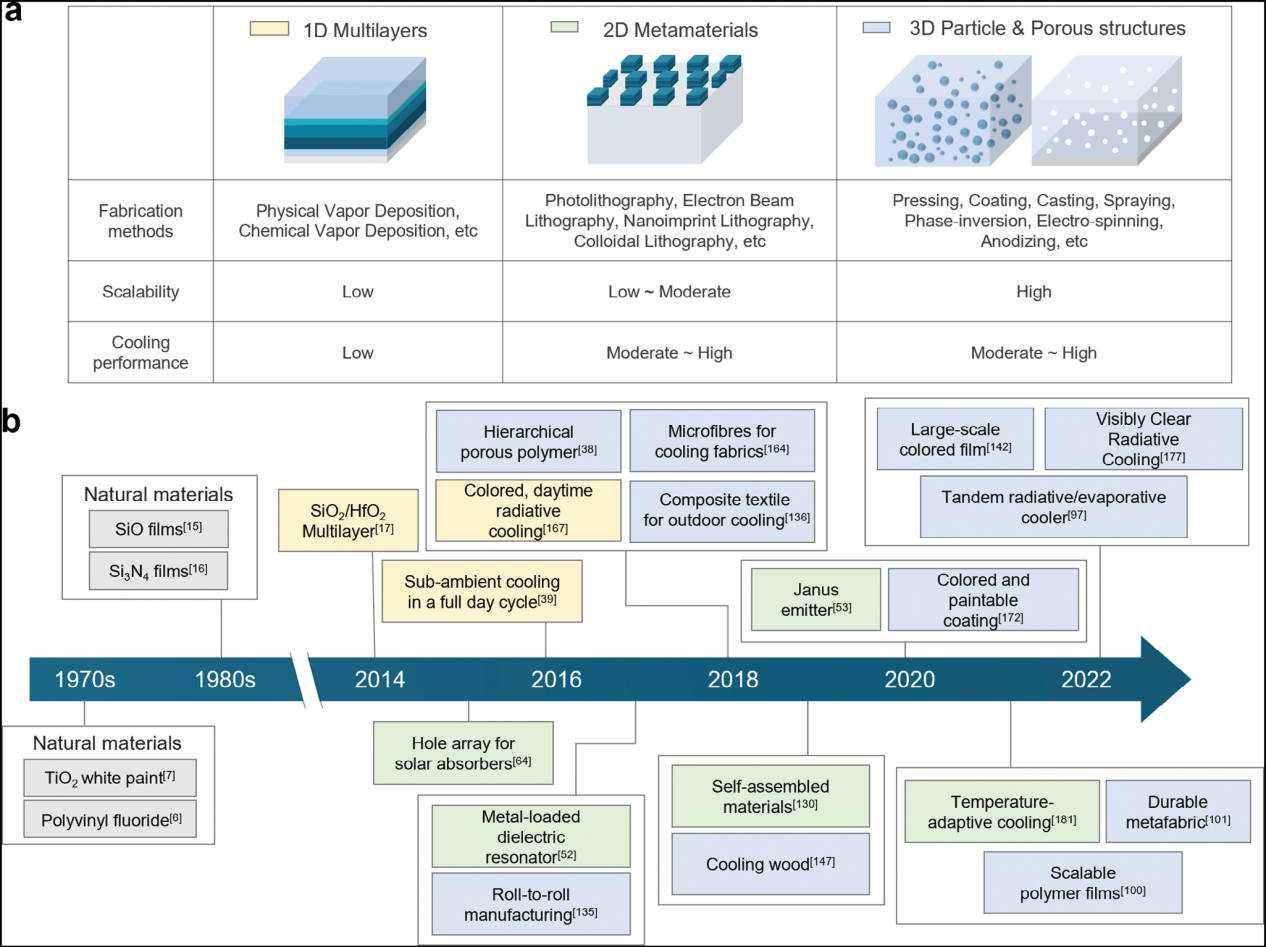
Overview of the development of RC. a) Structural designs for RC and their fabrication methods, b) timeline of the development of RC technology, categorized by structural designs. Gray: natural materials, yellow: 1D multilayer structure, green: 2D metamaterials, blue: 3D particle and porous structures.

Radiative coolers with different structural configurations have been realized by exploiting a variety of micro‐ arnd nano‐processing techniques, including thin‐film deposition, lithography, and effective‐medium formation (**Figure** **2**a). In this section, we provide an overview of recent advances in daytime RC from the point of view of fabrication method (Figure 2b). After thoroughly reviewing various fabrication techniques to realize radiative coolers, we then examine potential fabrication methods for practical uses.

### 3.1 1D Fabrication

Early studies on daytime RC attempted to extract thermo–optical properties from multilayer structures. Due to their simple design configurations, multilayer structures can readily be fabricated by facile methods. Typically, 1D multilayer structures can be fabricated by sequential thin film processing. Thin film processing is divided into: 1) surface‐processing techniques that either increase the effect or impart functionality to the base material and 2) coating or depositing other substances on the base material. Overall, in this review, thin‐film processing entails coating and deposition into the base material. Multilayer structures can be realized by growing metal and dielectric films on substrates by physical vapor deposition (PVD)^[^ ^103^ ^]^ and chemical vapor deposition (CVD).^[^ ^104^, ^105^ ^]^

**Figure 3:**
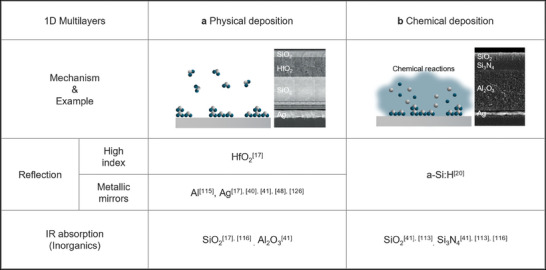
Fabrication methods for 1D daytime radiative coolers. a) A thin film deposition is started by ripping target base material off with physical force. b) A thin film is grown on the substrate by reacting between vapor‐phase precursors and reactive gas. Panel (a): Adapted with permission.^[^ ^17^ ^]^ Nature Springer. Panel (b): Adapted with permission.^[^ ^41^ ^]^ ACS Publications.

PVD is a set of techniques that deposit target materials onto a substrate by either evaporation or sputtering under a high vacuum (**Figure** **3**a). PVD can be classified into three main methods: electron‐beam evaporation,^[^ ^106^ ^]^ sputtering,^[^ ^107^ ^]^ and thermal evaporation^[^ ^108^ ^]^ depending on the method used to produce the film. PVD can provide a stable process with a simple mechanism to deposit target materials by physical force; thus, it enables low‐temperature deposition under high vacuum and produces high‐purity thin film regardless of the substrate.

PVD has been widely employed in the field of RC to deposit metallic mirrors, such as Ag, Al, and various inorganic materials, onto both commercial substrates and flexible polymer films.^[^ ^109^, ^110^ ^]^ These deposited Ag and Al metal mirrors are capable of reflecting the solar spectrum, leading to a significant reduction in *P*_sun_, a major contributor to cooling flux loss. Moreover, PVD enables the deposition of IR absorptive inorganic materials including SiO_2_, Si_3_N_4_, and Al_2_O_3_ within the same chamber, offering versatility and convenience for RC research. Notably, the pioneering work on sub‐ambient daytime RC utilized multilayer structures composed of dielectrics and metal mirrors, realized through electron beam evaporation (Figure 3a).^[^ ^17^ ^]^ The simplicity and ease of fabrication offered by PVD have spurred numerous research efforts, propelling advancements in the field of RC. However, the need for high vacuum increases the equipment's complexity, and the growth of mean free path (MFP) of evaporative particle can form uneven step coverage, which can yield low adhesion and poor uniformity.

CVD, on the other hand, uses chemical reactions to grow thin films of the target materials on the substrate from vapor‐phase precursors (Figure 3b). CVD can be classified according to the precursor‐decomposition methods, such as: thermal CVD,^[^ ^111^ ^]^ plasma‐enhanced (PE) CVD,^[^ ^112^ ^]^ and atomic layer deposition(ALD). CVD allows large‐area film growth with adequate step coverage by a series of reactions, including diffusion, adsorption, chemical reaction, and desorption over the entire area at a low vacuum. Consequently, CVD allows a more cost‐effective fabrication option compared to PVD, which necessitates the use of a high‐vacuum system. In addition, CVD has high versatility as it enables the growth of various thin films, including ceramics and semiconductors, by utilizing modified precursors. This enables the continuous deposition of several promising inorganic materials for daytime RC, such as SiO_2_, Al_2_O_3_, and Si_3_N_4_, using CVD. In addition, the modulation of precursors and established reactant gases in CVD allows the growth of materials with different absorption peaks, thereby increasing absorption across the broad IR spectral range. Therefore, several multilayer structures for sub‐ambient daytime RC, such as alternating SiO_2_/Si_3_N_4_,^[^ ^113^ ^]^ and a thermal‐emissive polymer with a CVD‐grown high‐/low‐refractive‐index Bragg mirror,^[^ ^20^ ^]^ have been developed using CVD. In addition, by using both PVD and CVD techniques, a radiative cooler with a long lifespan consisting entirely of inorganic materials has also been developed (Figure 3b).^[^ ^41^ ^]^ In a typical CVD process, the vacuum level and temperature should be carefully controlled to adjust the MFP and reaction rate to yield uniform film growth. However, operating at high vacuum results in a longer MFP and compromises the step coverage; while, low vacuum conditions can lead to the overhang phenomenon. It can be overcome by increasing the fabrication temperature at low vacuum to increase the particle mobilities in the film, but the use of low vacuum is subject to impurity problems. Moreover, the use of high‐temperature processes poses limitations when working with flexible polymers.

ALD can be considered to be a kind of CVD process and can be used to fabricate radiative coolers with high‐purity layers.^[^ ^58^ ^]^ ALD uses a vapor‐phase precursor and a reactive gas alternatively injected to grow atomic layers per cycle, and the repetition of this process forms a film layer. ALD allows formation of uniform step coverage with outstanding adsorption characteristics compared to other processes. By using ALD, the film thickness can be adjusted precisely; however, the atomic‐scale deposition has an extremely slow deposition rate. Consequently, it may not be suitable for growing thick films that are required to have sufficient IR absorptive capabilities over a large area for effective RC.

1D multilayer structures promoted initial RC studies with simple designs and fabrication methods. Gentle et al.^[^ ^114^ ^]^ utilized commercial reflector films, the ESR film produced by 3 M, with a reflectivity of up to 98%, as mirrors for a scalable daytime RC. Likewise, commercial polymer films with thicknesses of 50–100 µm have demonstrated comparable cooling performance to those fabricated in the laboratory when deposited with reflectors for use in daytime RC. The use of these price‐competitive reflectors or polymer films presents a promising opportunity for the commercialization of daytime radiative coolers. Despite their simple designs, multilayer structures have posed challenges in real‐world applications due to the limited degrees of freedom and the need for reflectors. These disadvantages led researchers to explore other structures of either 2D for enhancing cooling effects or 3D for scalability.

### 3.2 2D Fabrication

**Figure 4:**
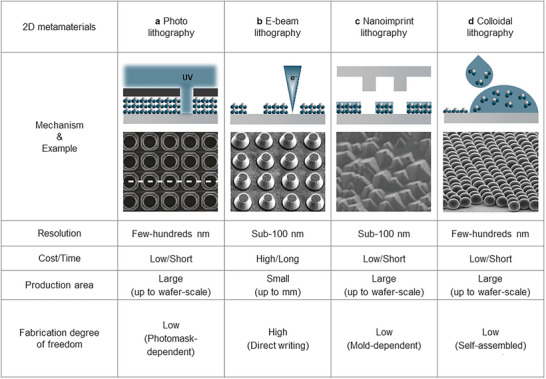
Fabrication methods for 1D and 2D daytime radiative coolers. Top–down lithography engraves bulk materials into microstructures and nanostructures. Representative categories top–down lithography use a) UV and b) electron beams. Bottom–up lithography stacks base materials or particles on the substrate by filling or assembling. Representative mechanisms of bottom–up lithography are c) imprint and d) self‐assembled colloidal. Panel (a): Adapted with permission.^[^ ^62^ ^]^ National Academy of Sciences. Panel (b): Adapted with permission.^[^ ^51^ ^]^ Wiley. Panel (c): Adapted with permission.^[^ ^126^ ^]^ Wiley. Panel (d): Adapted with permission.^[^ ^130^ ^]^Wiley.

By leveraging recent advances in nanofabrication techniques, researchers have successfully expanded the exploration of more intricate 2D structures, unlocking additional degrees of freedom and effectively enhancing cooling effect. These advancements have resulted in the creation of various 2D photonic structures that exhibit unprecedented light–matter interactions.^[^ ^115^ ^]^ These artificial 2D structures can be optimized through a series of designs to possess ideal RC optical characteristics, featuring high reflectance of the solar spectrum, along with selectively enhanced AW emission properties.^[^ ^54^ ^]^ The methods of forming 2D micro‐/nano‐structures can be categorized into top–down and bottom–up approaches. Top–down lithography forms the pattern by using a light source^[^ ^116^ ^]^ or electron beam^[^ ^117^ ^]^ to selectively remove parts of a bulk piece of material (**Figure** **4**a,b). Bottom–up processes stack small materials to form bulk nanostructures (Figure 4c,d).

Top–down approaches have been widely used because they offer high fidelity and controllability. Photolithography is a commonly used top–down fabrication approach in which a light source is projected through a mask onto a photoresist (PR), resulting in the formation of hard mask pattern. Subsequently, the exposed areas are etched away, leaving behind the desired pattern.^[^ ^116^ ^]^ It can be widely used to form the pattern on the substrate or through the post‐process such as the deposition process, which can form a certain pattern composed of target materials. To achieve high‐efficiency RC, promising inorganic materials can be processed using photolithography, enabling precise fabrication of the following microstructures that induce selective IR absorption.

RC has been achieved using an array of holes patterned by photolithography in glass (Figure 4a).^[^ ^62^ ^]^ A porous structure was formed by UV exposure through a square grid metal mask to form a PR mask. Then, the PR pattern was isotropically etched to form a periodic hole pattern. The periodic hole pattern induced high IR emission due to the impedance matching between the structure and the surrounding air. Subsequently, 2D pillar structures have been developed having similar optical properties to those of the hole array.^[^ ^118^, ^119^ ^]^ Inspired by natural creatures using photonic crystal to achieve RC, researchers have also explored the use of biomimetic photonic crystals as efficient radiative coolers. Hence, micro‐pyramid structures that mimic natural creatures for daytime RC have been achieved using both photolithography and chemical etching processes.^[^ ^56^, ^57^, ^120^ ^]^ The introduction of isotropic wet‐etching allows the realization of complex structures that are difficult to fabricate using only photolithography. However, photolithography relies on projection of light through a mask; so, the structures are highly dependent on the mask; this dependence imposes a constraint on the degree of freedom of structures that can be fabricated by the method.

Electron beam lithography (EBL) does not use a mask, thereby, alleviating the constraint associated with mask dependence. EBL uses a concentrated electron beam to scan an E‐beam‐sensitive resist and writes patterns directly.^[^ ^117^ ^]^ EBL allows the generation of features down to < 10 nm by utilizing a nanometer‐sized focused beam to scan along a user‐specified path. EBL has been used to write a conical shape of metal–dielectric alternating layers serving as a broadband IR absorber for RC (Figure 2b).^[^ ^51^ ^]^ This conical shape was fabricated by exploiting the physical phenomenon between sidewall deposition and lift‐off.^[^ ^121^ ^]^ Subsequently, a rectangular resonator composed of a metal–dielectric bilayer had been utilized to achieve additional thermal emission.^[^ ^52^ ^]^ In addition, EBL fabrication was also used to demonstrate smart cooling.^[^ ^122^ ^]^ A phase‐change material (e.g., VO_2_) was patterned on several dielectric layers, achieving temperature‐adaptive cooling. However, despite its high resolution, EBL is highly expensive and has low throughput due to its serial scanning nature, significantly limiting its practicality. Therefore, it has often been employed in the laboratory‐scale research to propose and demonstrate new structures for RC.

On the other hand, bottom–up approaches use materials to stack up using physical and chemical interactions and have been introduced for scalable RC fabrication. These methods can be easily scaled up to large areas by assembling small particles to form the bulk structures. Bottom–up approaches to realize 2D structures for RC have mainly been reported using nanoimprint lithography (NIL) and colloidal lithography (CL).

NIL creates patterns by placing a polymer resin into a mold and curing it under pressure.^[^ ^123^, ^124^ ^]^ NIL has been exploited for a scalable fabrication with high throughput due to the repeatable uses of molds. So far, NIL that uses various forms of resin, including promising inorganic‐nanoparticle‐embedded resins, has been reported, and it is being developed to a highly versatile process technology due to its high throughput.^[^ ^125^ ^]^ Highly efficient RC has been obtained by using a randomly‐distributed pyramidal inorganic structure that is imprinted from a slurry state and followed by post‐annealing (Figure 4c).^[^ ^126^ ^]^ Similarly, a disk array RC has also been successfully realized by exposing photocurable resin on a flexible mirror film to UV light.^[^ ^127^ ^]^ Notably, this fabrication method is compatible with a flexible polymer substrate because the method does not require high temperature or a chemical process. In addition, micro‐/nano‐structure can be transferred through a single‐step, high‐speed production process, indicating the potential for the commercialization of 2D RC.

CL is another approach to fabricate periodic arrays.^[^ ^128^, ^129^ ^]^ CL exploits the surface–energy interaction between spherical particles and a functionalized substrate. A suspension of spherical particles dispersed in a hydrophilic substrate self‐assembles to form a hexagonal array as the solvent evaporates. CL has been used to realize a simple radiative cooler that has a monolayer of microspheres (Figure 4d).^[^ ^130^ ^]^

Other bottom–up approaches have also been used to fabricate various 2D structures for RC.^[^ ^131^, ^132^, ^133^, ^134^ ^]^ Anodizing uses an electrochemical reaction and is typically used to produce anodic aluminum oxide (AAO) templates.^[^ ^131^, ^132^ ^]^ By using two‐step electrochemical anodization, a periodic and porous AAO template for RC has been developed.^[^ ^132^ ^]^ In addition to the AAO template, the AAO cooling effect has been further improved by coating it with a layer of ultra‐thin inorganic film grown by ALD. The emitter was then transferred to the Al substrate to reflect the solar spectrum, and thereby, operated under direct sunlight. Similarly, a two‐step anodization process has been used to fabricate a self‐aggregated AAO formed by capillary forces.^[^ ^133^ ^]^ A colorful radiative cooler has been obtained using a bioinspired array of SiO_2_ that was realized using a self‐assembled silver island structure.^[^ ^134^ ^]^

Although 2D fabrication can exhibit dramatic improvement in cooling performance via interesting structures, their intricate fabrication process may lead to increased fabrication costs per unit area. This poses a challenge when it comes to the commercialization of 2D radiative coolers with high performance.

### 3.3 3D Fabrication

3D structures such as particles and voids in media are easy to implement using high‐throughput fabrication methods and have been actively used in effective daytime radiative coolers. These structures induce a strong optical scattering effect and can be used to achieve high solar reflection. 3D radiative coolers can be made from materials compatible with high‐throughput fabrication methods and equipment; and are therefore, the most promising candidates for large‐scale applications. Methods to fabricate 3D radiative coolers can be divided into a bottom–up approach that mixes particles (Section 3.3.1) and a top–down approach that creates voids in the matrix (Section 3.3.2). Fabrication of particles or voids forms the effective medium by adding particles to a matrix or removing them from it.

#### 3.3.1 Particle Matrix

**Figure 5:**
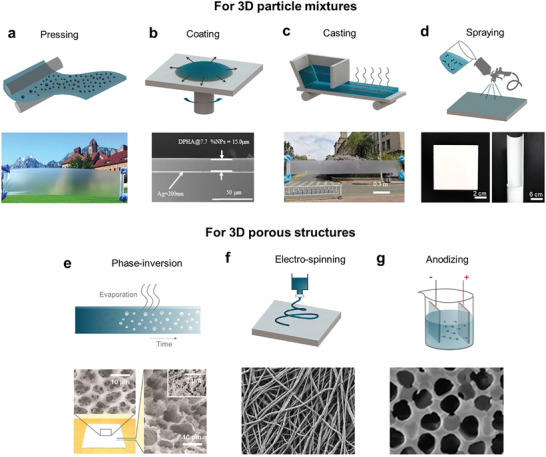
Fabrication methods for preparing 3D structures for daytime RC. a) In pressing, a uniform film is molded out by continuous press equipment. b) In coating, a thin film is formed by centrifugal force induced by chuck rotation. c) In casting, a liquid or slurry is poured into a mold to form a shape. Casting methods can be classified as natural spread or machine‐driven. d) In spraying, a jet is used to spread slurry onto a free‐curved surface at high speed. e) In phase inversion, porosity is achieved by randomly‐distributed voids that form as a result of a difference in solvent volatilization rates. f) In electrospinning, an electrically‐charged polymer solution spun through a jet forms a fiber network on a collector. g) In anodizing, a reaction to anodic voltages grows an oxide film on the metal in an electrolyte solution. Mainly, in RC research, anodizing is applied to fabricate porous anodized aluminum oxide (AAO) templates. Panel (a): Adapted with permission.^[^ ^135^ ^]^ AAAS. Panel (b): Adapted with permission.^[^ ^76^ ^]^ Elsevier. Panel (c): Adapted with permission.^[^ ^140^ ^]^ Elsevier. Panel (d): Adapted with permission.^[^ ^87^ ^]^ ACS Publications. Panel (e): Adapted with permission.^[^ ^38^ ^]^ AAAS. Panel (f): Adapted with permission.^[^ ^102^ ^]^ Nature Portfolio. Panel (g): Adapted with permission.^[^ ^146^ ^]^ Elsevier.

Various methods have been used to convert a particle mixture into the desired form. Pressing uses mechanical pressure to fabricate uniform structures such as film or plate; the method is highly scalable fabrication of devices (**Figure** **5**a) and achieves scalable manufacturing of several radiative coolers. One example^[^ ^135^ ^]^ is a bi‐layer thermoplastic polymer that contains randomly‐dispersed inorganic particles and is coated on a metal mirror (Figure 5a). A composite that contains the particles is pressed between heated upper and lower rolls resulting in a film with uniform thickness. Another similar fabrication method involves the use of a melting press to produce a film‐type radiative cooler made of inorganic–organic composite.^[^ ^136^ ^]^ The scalability of such techniques drives research to find new functionalities and applications, such as a mass‐produced inorganic and polymer mixtures film^[^ ^137^ ^]^ that becomes a greenish color after pressure molding with heated rolls; the colored cooler can be used as an artificial lawn.

High‐speed coating has also been proposed as a method to produce RC films. Spin‐coating is a fast and simple technique to coat uniform films on a lab scale. Early research^[^ ^40^ ^]^ demonstrated a thin film for RC by spin‐coating a polymer onto a mirror substrate; then, curing the polymer. The productivity of the process could be increased by using simple coatings by mixing a polymer, inorganic particles, and a UV‐curable resin. This composite was spin‐coated on a mirror; then, cured by UV exposure; however, this curing process partially negated the advantage of the rapid fabrication process. To reduce the number of fabrication steps, composites of two inorganic particles in an acrylate binder could produce films without the need for post‐processing^[^ ^76^ ^]^ (Figure 5b). The productivity was increased by omitting the UV‐exposure process. Other coating methods have also been used to fabricate radiative coolers. For example, a RC film was developed by using dip‐coating, which exploited the surface tension between the precursor solution and the sample.^[^ ^48^ ^]^ Improving the scalability of the process was important to drive potential large‐scale applications but should be done without degrading the effectiveness of radiative coolers; hence, a composite film containing hierarchically inorganic core–shell particles has been developed for high effectiveness, obtained by high‐temperature sintering of thin inorganic film.^[^ ^85^ ^]^ Additional backscattering resulting from the core–shell shape contributes to increased cooling effect. Despite its high scattering efficiency, the high‐temperature treatment process can act as a limiting factor for the application and productivity of flexible substrates.

Composites that include particles can also be applied by casting a slurry; the inorganic particles are dispersed within a composite material. This method uses molds to process slurries into the desired shape and has been presented for mass production. These advantages suggest the potential for the application of RC composite in various exterior materials. Therefore, a RC film is produced^[^ ^73^, ^74^ ^]^ by casting slurries composed of polymer‐dissolved solvents with added inorganic particles; ultrasonication promotes the distribution of the particles within the matrix to form a slurry that has effective optical properties. The slurry cast on the mold is converted to a free‐standing film by removing the air bubbles and the solvent. Using tools to disperse the slurry can further accelerate productivity.

Tape casting, also called blade coating, spreads the slurry through a certain edge to form films. A specific amount of slurry is poured onto a flat tape; then, a blade at a certain height above the flat plate spreads the slurry forward to form a film of a certain thickness (Figure 5c). A free‐standing film can be obtained by drying slurry and removing the tape. The possibility of large‐area fabrication by blade coating is first demonstrated^[^ ^138^ ^]^ using a pre‐polymer on a mirror. This possibility has stimulated various subsequent studies. For example, a cooling film was demonstrated using a double layer of inorganic–organic composite that had been deposited by tape casting.^[^ ^75^ ^]^ Similarly, a cooling film was achieved using screen‐printing. These advances suggest that slurries may be widely applicable.^[^ ^139^ ^]^ A radiative cooler with self‐cleaning functionality, which avoids degraded cooling performance due to surface dust or debris, is obtained by coupling functional groups to hierarchical‐size spherical inorganic particles to induce superhydrophobicity.^[^ ^140^ ^]^ (Figure 5c).

Spray coating can directly coat materials regardless of substrates and surface curvatures (Figure 5d). Several spray‐coating methods are reported for practical RC. For example, films that increase optical scattering are obtained by spraying crystal particle‐based suspension on a mirror.^[^ ^67^ ^]^ In addition, cooling coatings are obtained by spraying an inorganic geopolymer^[^ ^87^ ^]^ (Figure 5d). Using periodically moving equipment during the spray process facilitates the uniform formation of inorganic particle films, thereby avoiding unexpected optical responses and achieving expected cooling performance approximating the design. Use of spraying to fabricate RC layers offers great advantages because it can use any type of slurry or matter.

#### 3.3.2 Void Matrix

Effective emissive composites with voids that can facilitate solar scattering can induce RC. Fabrication of porous matrix is easily scalable and can support mass production of radiative coolers. A representative process is phase inversion that generates voids by exploiting the discrepancy in the volatilization rates of inter‐solvents during polymerization of a matrix dissolved in solvents (Figure 5e). The mechanism of dissolving particles by solvents can also be regarded as a kind of phase inversion.

Porous RC films have been achieved using hierarchical pores formed by the different volatilization rates of solvents.^[^ ^38^ ^]^ A porous matrix with random‐sized voids can be formed depending on the solvent evaporation path in the polymer. The matrix can be easily fabricated by coating and drying. A subsequent study^[^ ^141^ ^]^ used self‐assembled multilayer polymer beads as templates to construct periodic porous structures. After the polymer was infiltrated into a template, an annealing process removed the beads to produce a flexible thermal emitter that included periodic voids. Similarly, porous cooling films had been fabricated by dissolving away granulated sugar that had been dispersed in water on the surface of the polymer.^[^ ^90^ ^]^ Recently, a combination of phase inversion method, blade coating, and a roll‐to‐roll process demonstrated scalable manufacturing of a radiative cooler at the meter scale.^[^ ^142^ ^]^

Fabrication schemes that use more than one material, such as mixing particles^[^ ^89^, ^92^, ^143^ ^]^ or adding functional materials for hybrid cooling,^[^ ^144^ ^]^ have been combined with the phase‐inversion method. For example, a hierarchical porous structure has been used for sub‐ambient RC. The structure can be fabricated by casting a polymer that contains dispersed nanoparticles in self‐assembled hexagonal beads to fabricate layered porous arrays.^[^ ^89^ ^]^ Subsequent etching of periodic microbeads and nanoparticles yields hierarchical porous arrays. Although two‐step etching achieves high cooling effect, the method has insufficient productivity. Therefore, alternative methods have been proposed that offer high production efficiency with simple processes.

Mixing particles that have high refractive index into a phase‐inversion precursor can yield a complex porous‐particle structure.^[^ ^143^ ^]^ The hierarchical voids and additional backscattering of high‐index particles contribute to the increase in solar reflection. A strategy that exploits the particle's gravitational sedimentation and phase inversion can achieve a porous‐particle bilayer in a single process;^[^ ^92^ ^]^ this approach yields devices that have higher cooling effect than others that had been produced at similar fabrication speeds. RC has also been achieved using hydrogel‐porous polymer;^[^ ^144^ ^]^ this structure utilized an additional cooling effect caused by the evaporation of moisture absorbed by the hydrogel and could substantially increase the passive cooling effect. The hybrid structure was manufactured using two‐step casting and phase inversion; and therefore, could have high productivity.

Electrospinning shoots electrically‐charged polymer solutions through jets onto a collector to form a fiber network. This method has been used to fabricate porous‐arranged RC fabrics (Figure 5f). Use of a phase inversion precursor in the polymer solutions and electrospinning can acquire additively hierarchical porosity inside the fibers.^[^ ^96^ ^]^ In addition, two‐step processes of electrospinning and emulsion deposition yield a flexible hybrid membrane radiative cooler, and the production is highly effective and scalable.^[^ ^71^ ^]^ Several radiative coolers use natural fibers of silk and further process them. For example, nano‐processed silk has been produced using a scalable coupling‐reagent‐assisted dip coating method.^[^ ^102^ ^]^ Tetrabutyl titanate was used as a coupling reagent that connects the inorganic nanoparticles of Al_2_O_3_ to natural silk, and this combination significantly increases the RC effect. Similarly, artificially‐restructured micro‐scale/nano‐scale silk was produced by electrospinning, and effectively scattered sunlight.^[^ ^145^ ^]^ These methods to fabricate RC fiber are compatible with commercial textile manufacturing equipment and are promising low‐cost, high‐throughput manufacturing methods. Use of evaporation cooling has also been used in fabrics. Functionalized hydrogels and porous fibrous bilayers have been combined to form a hybrid porous network by combining a phase‐separation precursor and hydrogels that contain random pores made by freeze‐thawing and fiber electrospinning.^[^ ^97^ ^]^ Another bilayer fabric that uses hierarchically‐sized particles was produced using a two‐step electrospinning process.^[^ ^98^ ^]^ The fabricated fabric was treated in hydrophilic solution to promote additional evaporative cooling. Hierarchically hollow core and shell cooling fibers have been formed by coaxial electrospinning that uses two types of phase separation precursors.^[^ ^99^ ^]^ Further, an existing electrospinning technique has been combined with a roll‐to‐roll collector to produce nanofibers that had constant thickness and could be produced on meter scale.^[^ ^100^ ^]^ Melt spinning had also been used to produce cooling fibers that were made from polymer pellets that contained inorganic particles injected by melt extrusion mixing.^[^ ^101^ ^]^

Other conventional fabrication methods to achieve RC have used various materials that incorporate porous structures. For example, a ball‐milling method can form uniform polymer micro‐clusters to increase solar reflection.^[^ ^91^ ^]^ Two‐step anodization can increase the porosity of an AAO template (Figure 5 g).^[^ ^146^ ^]^ Delignifying and repressing natural wood, a structural material with mesoporous cellulose was developed and showed highly effective RC.^[^ ^147^ ^]^ Recently, a new approach utilizing 3D direct printing had been applied to develop radiative coolers. The primary technique of 3D printing involved introducing materials into the supply mechanism and extruding them through a nozzle. For example, it could be utilized as a mixed resin consisting of inorganic particles and solvents to enable IR emission and phase conversion.^[^ ^148^ ^]^ Thanks to this intuitive fabrication mechanism, it was possible to rapidly produce a wide range of shapes without being constrained by pre‐existing structures. Likewise, porous structures could be readily fabricated using a variety of materials and fabrication methods; and therefore, have numerous potential practical uses.

### 3.4 Expected Method

With the support of micro‐/nano‐processing, RC technology has achieved rapid advancements in cooling effectiveness and scalability and has been developed in the direction of improving cooling efficiency and fabrication productivity. The research paradigm, which is shifting toward commercialization, can be well‐observed from big (3 M, Gore‐tex Corp., etc.) and emerging venture companies (Radi‐cool, SkyCool System, Foel Inc., etc.) and market movements. RC technology is attracting substantial interest from diverse communities as a promising energy‐saving technology that has high feasibility. However, actual commercialization requires solutions to some remaining challenges. First, the RC effect is generally subject to the material's inherent optical properties. Hence, efforts should be made to develop and optimize materials. Second, to secure economic feasibility, RC technology can be implemented in real‐life only after development of facile fabrication methods that yield a reasonable cooling effect. Recently, optimized material technology in the field of photonics has been reported. Effective manipulation of light requires sub‐wavelength structures that have a high refractive index and low loss. For example, internal chemical reactions can be controlled by adjusting CVD conditions control to develop low‐loss amorphous silicon, which is transparent in the visible regime.^[^ ^149^ ^]^ This technology can be achieved by controlling the process conditions with existing equipment, and the method can be applied to different materials to seek optimized optical characteristics. Currently, these technologies have been applied to several materials and process technologies and have prompted subsequent studies.^[^ ^150^, ^151^ ^]^ In addition, this approach of controlling optical properties through process manipulation is not limited to CVD. For instance, the glancing angle deposition enables the development of novel materials. It can create porous films reducing solar absorption through scattering or enabling selective radiation based on their structure. These advancements have potential applications in the field of RC. The recent attempt to enhance light confinement by ALD‐deposited high‐refractive‐index ultrathin film on structured polymer, enabling control of the optical properties of the entire 12‐inch medium, has been reported.^[^ ^152^ ^]^ This strategy applicable to large areas can be utilized in a manner that contributes to enhancing cooling efficiency by using a novel approach; while, utilizing existing fabrication processes. Alternatively, embedding of TiO_2_ NPs, which have high refractive index with low loss can be dispersed in a UV‐curable resin to yield an effective medium,^[^ ^125^, ^153^ ^]^ and the optical properties of the materials can be modulated by adjusting the volume fraction of particles. This fabrication process can replace the high‐cost lithography and ALD processes by using the inexpensive nanoimprint and coating processes. The utilization of inorganic particle‐embedded resin in patterned roll‐to‐roll or roll‐to‐plate processes also offers the potential for the mass production of patterned 2D arrays. Depending on their design, enhanced selective reflection and emission characteristics can be induced, thereby enabling the commercialization of high‐efficiency RC films. We believe that these upcoming technologies are integrated with advanced technology, which may provide a breakthrough toward the commercialization of RC.

## 4 Potential Functionalities and Applications of RC

Despite its short history, the rapid advance of RC technology has promoted its implementation into real‐world applications and even commercialization. However, for practical applications, other aspects of radiative coolers besides the cooling effect and fabrication methods should also be considered. Such aspects include material qualities, visual appearance, switching capability, durability, and compatibility with other fields such as photovoltaic,^[^ ^62^, ^154^ ^]^ thermoelectricity,^[^ ^155^, ^156^ ^]^ and water harvesting.^[^ ^157^, ^158^ ^]^ In this section, recent efforts to address such practical issues are reviewed. First, radiative coolers that can be simply coated on the exterior walls in large‐scale and cooling textiles are summarized. Second, radiative coolers with aesthetic functions are discussed. The realizations of colored or transparent radiative coolers by controlling the spectra in the solar range are covered. Finally, temperature‐adaptive radiative coolers that provide the cooling effect only at high temperatures are reviewed.

### 4.1 Facile Techniques for Large‐Scale Exterior Coating and Textiles

**Figure 6:**
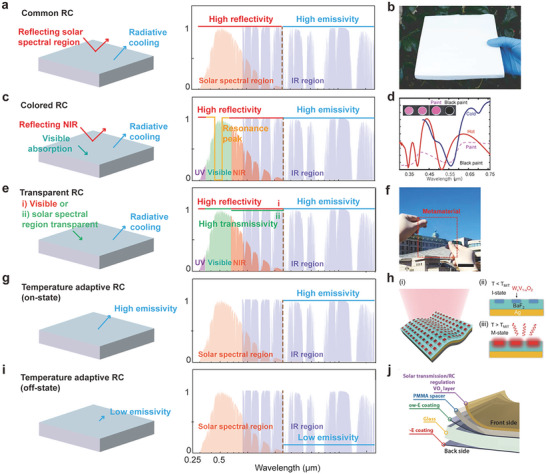
Photonic concepts and applications of RC. a) RC concept having high reflectivity in solar spectral regime and high emissivity in MIR regime. b) RC wood by engineered cellulose nanofibers. c) RC concept having resonance in visible regime for color generation and high reflectivity and emissivity in the other regime. d) Reflectivity spectra of two different RC films in visible regime. Even the two multilayer structures show similar color; the difference of thermal emissivity leads to significant gap of cooling effect. e) RC concept having high transmissivity: e‐i) only in visible regime or e‐ii) in solar spectral region and high emissivity in MIR regime. f) Transparent RC film by SiO_2_ aerogel nanoparticles randomly distributed in PDMS. The RC film shows high transparency in solar spectrum regime with high emissivity in MIR regime. g) RC concept having high emissivity in MIR region. h‐i) Schematic and h‐ii,iii) working mechanism of the temperature adaptive radiative cooler. Patterned array of V*_x_* V*_1_*_−_ *_x_* O_2_ blocks in a BaF_2_ layer on Ag film represents switchable cooling effect below and above the transition temperature as shown in right side spectra. i) RC concept having low emissivity in MIR region. j) Scalable temperature adaptive radiative cooler by simple solution process based on VO_2_/spacer/low‐E stacked film (inset figure) and its switchable emissivity spectra. Panel (b): Adapted with permission.^[^ ^147^ ^]^ AAAS. Panel (d): Adapted with permission.^[^ ^46^ ^]^ Springer Nature. Panel (f): Adapted with permission.^[^ ^177^ ^]^ Wiley. Panel (h): Adapted with permission.^[^ ^181^ ^]^ AAAS. Panel (j): Adapted with permission.^[^ ^182^ ^]^ AAAS.

To facilitate the implementation of the radiative coolers into practical applications, scalable manufacturing of the coolers, that is, particle–polymer composites coatings^[^ ^135^, ^159^ ^]^ and porous polymers,^[^ ^38^, ^160^, ^161^ ^]^ has been developed. These scalable methods have enabled the application of radiative coolers to large‐scale exterior walls. The radiative coolers in early studies have been designed to exhibit high reflectivity in all solar spectrum and high emissivity in the MIR regime to maximize the outdoor cooling effect (**Figure** **6**a). RC materials satisfying this concept have not only verified their potential to be used for exterior walls but can also be produced in various forms of coatings, paints, and blocks. Polymer mixed micrometer‐sized SiO_2_ spheres‐based high‐throughput and cost‐effective roll‐to‐roll method using polymethylpentene (TPX) demonstrated its superior flexibility and scalable fabrication ability that can cover the outdoor structure exterior.^[^ ^135^ ^]^ A paint‐like facile fabrication of hierarchically porous poly(vinylidene fluoride‐co‐ hexafluoropropylene) coatings improves the convenience of making surface coating due to the ease of spraying onto a wide range of surfaces.^[^ ^38^ ^]^ A RC wood block with excellent mechanical strength was also introduced by cellulose nanofibers (Figure 6b).^[^ ^147^ ^]^ The ease of manufacturing RC coatings, paints, and block‐type building materials has proven the potential for use in outdoor structures such as real buildings, as verified in the cooling performance evaluation of the actual warehouse size.^[^ ^162^ ^]^

Another main use of RC is a cooling textile,^[^ ^22^, ^163^, ^164^, ^165^ ^]^ which can be used for various purposes such as wearable devices and clothes. In the case of textiles, the common RC concept illustrated in Figure 6a is required because the surface temperature under direct sunlight is generally higher than the body temperature. Recently, a radiative cooler made by the nano‐processed silk was proposed by using a molecular bonding design strategy and scalable dip‐coating method. The stand‐alone nano‐processed silk demonstrated 3.5 °C sub‐ambient cooling under direct sunlight and the temperature reduction ≈8 °C compared to the natural silk, verifying its cooling potential.^[^ ^102^ ^]^ Another large‐scale woven fabric by hierarchical‐morphology design was reported.^[^ ^101^ ^]^ Randomly dispersed scatters throughout the fabric achieved high reflectivity of 92.4% with high emissivity of 94.5%. The test on a human body covered by woven fabric demonstrated that it could cool down ≈4.8 °C compared to common cotton fabric.

### 4.2 Coloration and Transparency

While the concept of RC is typically to control the radiative thermal load of outdoor structures on buildings, automobiles, and clothing, the visual aesthetic is also a significant aspect that cannot be ignored. As the aforementioned approaches perfectly reflect light in the visible range, controlling the thermal load in the colored structure is not achievable. The presence of a reflectance resonance in the visible region, which is inside the solar spectrum, allows a radiative cooler to exhibit color (Figure 6c). Including early studies showing the reflection of different peaks using metal oxides in the paint mixture,^[^ ^166^ ^]^ studies for esthetic properties of materials for daytime RC using optimized nanostructures, materials, and photoluminescence; have been reported.^[^ ^167^, ^168^, ^169^, ^170^, ^171^ ^]^ Various research has reported the realization of color by controlling resonance in the visible region using various nanostructures such as multilayers and particles.^[^ ^46^, ^167^, ^168^, ^172^, ^173^ ^]^ These nanostructures can lead to resonance by adjusting the spacer thickness or scattering properties from the distributed particles.

However, a tradeoff between color and cooling effect has arisen due to the cooling power loss from the resonance in the visible region.^[^ ^20^, ^167^ ^]^ Structural optimization for balance between the esthetic function and cooling capacity is inevitably a significant design criterion. Fortunately, the reflection spectrum and the representative color do not obey one‐to‐one correspondence. In other words, it is possible to design two radiative coolers that exhibit the same color but have different cooling effects. Therefore, the sophisticated design of the radiative cooler can bring out the realization of daytime RC while exhibiting an aesthetic effect. The example of a radiative cooler showing the same pink color broke the balance of color and cooling performance and provided a clue to find the optimal design (Figure 6d).^[^ ^46^ ^]^ Experimental results showed that two photonic multilayer structures having similar color could have different net cooling flux. The tunable range was from 680 W m^−2^ for all color to 866 W m^−2^, which can be 47.6 °C temperature difference under direct sunlight. This study demonstrated the significant potential of the colored radiative cooler.

The control of thermal load via spectral manipulation in a specific wavelength region also suggests the possibilities of transparent RC. Transparency also has a great impact on practical applications of RC such as vehicles and buildings. Based on this concept, various efforts to serve two ends between transparency and RC have been reported.^[^ ^20^, ^140^, ^174^ ^]^ The first visibly transparent radiative cooler was designed to have high transmissivity only in the visible spectrum while being reflective in the non‐visible solar range (UV and NIR region) and emits its thermal energy in the MIR region (Figure 6e‐i). A transparent radiative cooler based on multilayer structure of selective reflector and PDMS emitter layer was investigated.^[^ ^20^ ^]^ The selective reflector partially transmits visible light and reflects undesirable NIR region (from 0.74 to 1.4 µm). The top PDMS layer radiates thermal energy through the AW. Consequently, the maximum temperature reduction of 14.4 °C was achieved during the daytime. In addition to the effort to control the transmittance of only the visible region, a similar concept of transparent RC including the NIR region was reported (Figure 6e‐ii).^[^ ^174^, ^175^ ^]^

Solar cells are another good application for the transparency of RC.^[^ ^21^, ^176^, ^177^ ^]^ Solar cells operated under direct sunlight warm up naturally, which can affect reliability and efficiency.^[^ ^178^ ^]^ Therefore, optimized RC having maximized thermal emission and transparency to sunlight is required. It is ideal to exhibit high emissivity in the MIR region over 4 µm or more while maintaining high transmittance in the solar spectrum.^[^ ^21^ ^]^ Recently, visibly clear and flexible RC materials have been developed (Figure 6f) using randomly distributed silica aerogel microparticles in a silicone elastomer.^[^ ^177^ ^]^ The deployment in solar cells demonstrates effective suppression of the temperature increase under solar irradiation, thereby mitigating the performance degradation of solar cells due to the heating issues by satisfying the photonic concept of high transmittance in the overall solar spectral region.

### 4.3 Temperature Adaptive Regulation

Various research on RC has been developed based on passive photonic structures. However, it is difficult to judge that passive RC will always work if it has different environments and temperatures according to seasons or regions. At ambient temperature that does not require cooling, an active switchable RC concept, in which the cooling can be turned on and off, is important. The key concept of these active switchable radiative coolers is to control the emissivity of the MIR region. When the emissivity of MIR is high, the cooler is an on‐state because it can emit a lot of heat (Figure 6 g); whereas in the opposite case, it is an off‐state due to the limited thermal emission (Figure 6i). Since the theoretical proposal of the multilayer structure using a phase change material,^[^ ^47^ ^]^ various efforts toward such switchable RC systems have been followed.^[^ ^179^, ^180^ ^]^ These results here lead to new functionalities of RC and can potentially be used in a wide range of applications for the thermal managements of buildings, vehicles, and textiles,^[^ ^47^ ^]^ with advances in technology for practical use that can meet the photonics standpoint.

For the development of switchable radiative coolers, it is essential to find temperature‐responsive materials with a transition temperature near room temperature. While doped VO_2_ exhibits a transition temperature around room temperature, the desirable transition temperature in the indoor environment is still lower than this.^[^ ^47^, ^133^ ^]^ A temperature‐adaptive switchable radiative coating using W*_x_* V_1−_ *_x_* O_2_ was developed, and the possibility of achieving the desired transition temperature (≈22 °C) was demonstrated.^[^ ^181^ ^]^ This active RC material shows switchable thermal emissivity by photonically amplified metal–insulator transition from 0.20 to 0.90 for the ambient temperature changes lower than 15 °C and above 30 °C (Figure 6 h). Another problem to be tackled for practical use is that the materials for switchable RC are difficult to fabricate, especially in large scale. Recently, a thermochromic coating based on tungsten‐doped VO_2_‐PMMA/spacer/low‐E stack using a solution process was reported to satisfy the demands of switchable RC function in different temperature.^[^ ^182^ ^]^ The modulation ability of MIR emissivity of this coating can be adjusted by tuning the spacer thickness and VO_2_ weight ratio and doping. Simple solution process by coating giving different emissivity of 0.61 (at high temperature) and 0.21 (at low temperature) regulates RC automatically; while, maintaining visible region transparency and near‐infrared modulation (Figure 6j). These temperatures‐adaptive regulations of RC can be utilized in various applications throughout various climatic conditions.

## 5 Conclusion and Outlook

RC is a promising new direction in sustainable energy research. Here, we have reviewed progress in research on RC technology, from the early demonstrations to recent advances toward practical applications. We conclude by highlighting the challenges and future potentials of passive RC systems.

We note that some commercial companies such as 3 M and Gore‐tex Corp have successfully demonstrated the commercial viability of radiative coolers, showcasing the potential for real‐world applications. For instance, 3 M utilized multilayer structures for optical films that can cool the surfaces such as bus shelter roofs throughout the day and night. SkyCool Systems, founded in 2016, have developed a rooftop panel system that can be used as an add‐on to air conditioning and refrigeration systems leveraging the RC technology to reduce energy consumption. Other recently founded companies such as ChillSkyn have developed porous polymer coating and other forms of radiative cooling paint that can be easily applied to various surfaces including metal, plastics, and wood, showcasing its versatility in use. These emerging commercialized products are different from lab‐scale demonstrations in that they are particularly designed and tested to be implemented and produced in large‐scale offering efficient cooling solutions to various markets and industries. These applications include roofing in warehouses, factories, thermal power plants, and data centers as well as common transportations and vehicles used in shipping and deliveries.

However, in this shifting research paradigm, there are several challenges that need to be addressed to effectively promote the efficient commercialization and widespread implementation of other laboratory research on RC technologies.

First, low‐cost and ecologically‐benign fabrication methods need to be developed for large‐scale utilization. Although simple and scalable manufacturing methods have been evaluated, some are only compatible with specific materials and structures; further, some cause environmental problems, such as use of hazardous chemicals and emission of volatile organic compounds.^[^ ^92^, ^93^, ^183^ ^]^ To realize real‐world implementation of radiative coolers, researchers must find suitable materials while developing scalable, cost‐effective, and ecologically‐benign fabrication methods for commercialization. This involves optimizing fabrication techniques, reducing material costs, and streamlining production processes. Second, RC is highly sensitive to humidity and regional climate conditions. Humidity in the air significantly lowers the atmospheric transmission and degrades the heat dissipation of the cooler. Radiative coolers that are robust to surrounding atmospheric condition would greatly improve the efficacy of radiative cooling in many applications. In addition, the establishment of specific standards and regulations for RC technology will provide crucial support for its commercialization, ensuring defined performance metrics, safety guidelines, and overall quality and reliability. Last, integration of RC technologies into existing infrastructure poses another challenge. Radiative coolers need to be seamlessly integrated into buildings, cooling networks, and other relevant systems. Compatibility with different applications and effective integration strategies will be crucial to ensure smooth implementation and maximize the benefits of RC technology.

While challenges and limitations remain, recent work on functional RC suggests emerging research directions and prospective applications. As explained above, the effectiveness of a radiative cooler has almost reached its limit. To boost the effectiveness of a passive cooling system, a hybrid approach that uses both radiative and evaporative cooling can be used.^[^ ^184^, ^185^, ^186^, ^187^ ^]^ This approach could overcome the fundamental difficulties arising from humid atmosphere and significantly increase the efficiency of a passive cooling system by exploiting two different heat transfer mechanisms.

We anticipate that RC will be used in an increasing range of applications and will have considerable technological impact. Besides the functionalities reviewed in Section 4, development of radiative coolers with improved self‐cleaning and durability suggests promising applications for surfaces that require long‐term use.^[^ ^97^, ^188^, ^189^, ^190^, ^191^ ^]^ Combining RC concepts with various research in sustainable energy and thermal managing field such as photothermal^[^ ^192^ ^]^ and vapor condensation^[^ ^193^ ^]^ offers great functional integration of a device. Improving the availability and reliability of RC would be the next crucial step for the engineers and researchers.

Beyond simply increasing cooling effect, research in RC is now moving toward practical applications and integration with other thermal management systems. We believe that our review will inspire many researchers of nanotechnology, optics, energy, and other disciplines, and will propel this ecologically‐benign technology toward practical applications in energy saving.

## Conflict of Interest

The authors declare no conflict of interest.

## Supporting information

Supporting Information
