# Supplementary material for: Radiative Cooling for Energy Sustainability: From Fundamentals to Fabrication Methods Toward Commercialization
Source: Adv Sci (Weinh). 2023 Nov 10;11(2):2305067. doi: 10.1002/advs.202305067 (PMC10787071; doi:10.1002/advs.202305067)
Supplement: Supplementary file 1 — Supporting Information [file ADVS-11-2305067-s001.pdf]

ADVANCED SCIENCE
Open Access

Supporting Information

for *Adv. Sci.*, DOI 10.1002/advs.202305067

Radiative Cooling for Energy Sustainability: From Fundamentals to Fabrication Methods Toward Commercialization

*Sunae So, Jooyeong Yun, Byoungsu Ko, Dasol Lee, Minkyung Kim, Jaebum Noh, Cherry Park, Junkyeong Park and Junsuk Rho**

Supporting Information for

Radiative cooling for energy sustainability: From fundamentals to fabrication methods towards commercialization

Sunae So[1,2,3,†], Jooyeong Yun[2,†], Byoungsu Ko[2,†], Dasol Lee[2,4,†], Minkyung Kim[2,5], Jaebum Noh[2], Cherry Park[2], Junkyeong Park[2], Junsuk Rho[2,6,7,*]

[1]Graduate School of Artificial Intelligence, Pohang University of Science and Technology (POSTECH), Pohang 37673, Republic of Korea

[2]Department of Mechanical Engineering, Pohang University of Science and Technology (POSTECH), Pohang 37673, Republic of Korea

[3]Department of Electro-Mechanical Systems Engineering, Korea University, Sejong 30019, Republic of Korea

[4]Department of Biomedical Engineering, Yonsei University, Wonju 26493, Republic of Korea

[5]School of Mechanical Engineering, Gwangju Institute of Science and Technology (GIST), Gwangju 61005, Republic of Korea

[6]Department of Chemical Engineering, Pohang University of Science and Technology (POSTECH), Pohang 37673, Republic of Korea

[7]POSCO-POSTECH-RIST Convergence Research Center for Flat Optics and Metaphotonics, Pohang 37673, Republic of Korea

Supplementary Note 1. Fundamentals of passive radiative cooling

Supplementary Note 2. Heat transfer

Supplementary Note 1. Fundamentals of passive radiative cooling

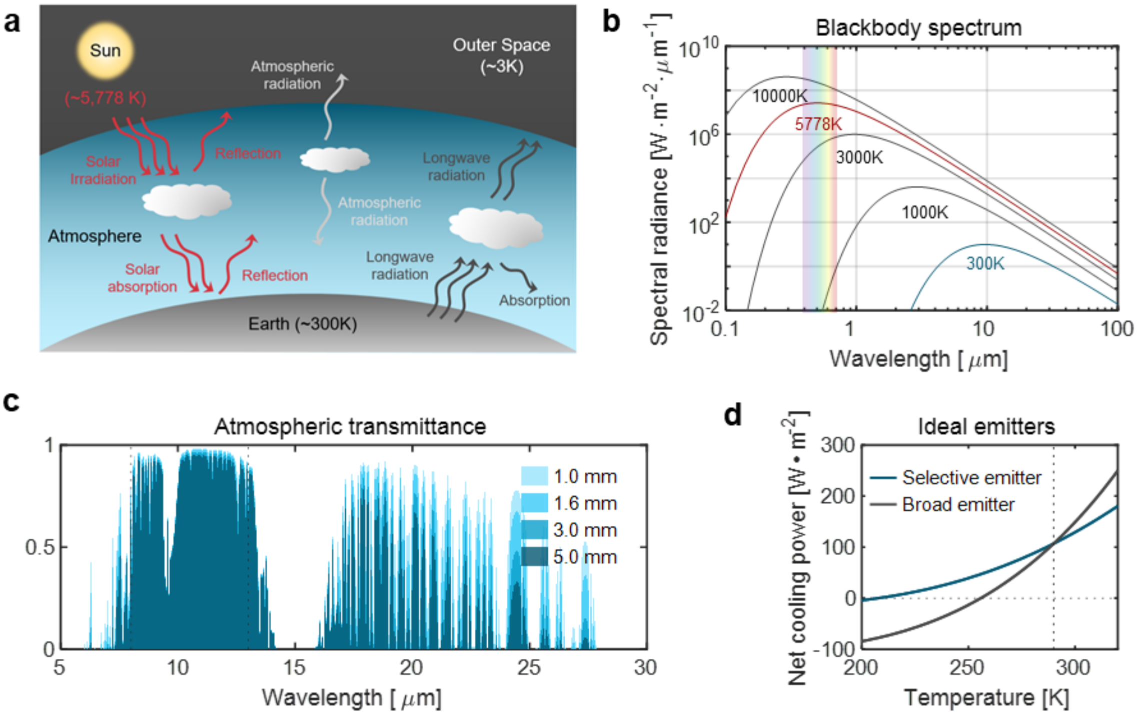

Supplementary Figure 1 | Fundamentals of radiative cooling a | Energy exchange processes between the Earth, atmosphere, and outer space. **b** | Spectral irradiation of blackbodies with various temperatures. **c** | Modeled atmospheric transmittance for different water vapor column (data were taken from Gemini observatory[1]). **d** | Calculated net cooling power of two ideal radiative coolers of the selective emitter and the broadband emitter at different temperatures in a typical clear sky in mid-latitude conditions.

Radiative cooling occurs by the energy exchange among the Earth, the Sun, and the universe. Any object with finite temperature continuously radiates thermal energy (Supplementary Box1); therefore, the Earth, the Sun, and the universe, with surface temperatures of around 300K, 5800K, and 3K, respectively, radiate thermal energy in the form of electromagnetic waves[2]. The Earth's temperature is determined by the balance between energy absorbed from the Sun (insolation) and energy emitted to the universe, modified by energy absorption by the atmosphere.

Consider energy exchange from an insulated surface to the universe through a clear sky (Supplementary Figure 1a). The net cooling power of the surface is the balance of energy flows of the absorption of insolation and atmospheric radiation, the emission of the surface, and the other heat losses. Considering all energy exchanges, the net cooling power $P_{net\_cooling}$ is given by[3]

$$P_{net\_cooling} = P_{rad} - P_{atm} - P_{solar} - P_{non-rad}, \quad (1)$$

where P_{rad} is the thermal radiation power emitted by the surface, P_{atm} is the atmospheric radiation absorbed by the surface, P_{solar} is the solar radiation absorbed by the surface, and $P_{non-rad}$ is an additional energy loss arising from the non-radiative heat transfer between the surface and the ambient. Each power term will be discussed in detail in the following subsections, then strategies to achieve maximum radiative cooling will be presented.

Thermal radiation from the surface

At temperature *T*, the total radiation power $P_{rad}(T)$ from a surface can be calculated by integrating thermal emission over all wavelengths and directions:

$$P_{rad}(T) = A_r \int_0^{\infty} \int_0^{2\pi} \int_0^{\frac{\pi}{2}} \varepsilon_r(\lambda, \theta, \phi, T) I_B(\lambda, T) \cos\theta \sin\theta \, d\theta d\phi d\lambda, \quad (2)$$

where A_r is the area of the radiating surface, $\varepsilon_r(\lambda, \theta, \phi, T)$ is emissivity of the surface at wavelength λ, zenith angle θ and azimuth angle ϕ at surface temperature *T*, $I_B(\lambda, T)$ is the thermal radiation of a blackbody (Supplementary Figure 1b and Supplementary Box1). The effects of T and ϕ are negligible compared to the effects of λ and θ. Therefore, we assume that the emissivity depends only on λ and θ (i.e., $\varepsilon(\lambda, \theta)$).

Atmospheric radiation

The atmosphere that surrounds Earth is composed of various gas molecules, including nitrogen N_2, oxygen O_2, water vapor H_2O, argon Ar, and carbon dioxide CO_2. These molecules absorb energy (solar or radiated from the earth and environment) then reradiate it (i.e., electromagnetic waves) at ambient temperature T_{amb}. Thus, the spectral intensity of atmospheric thermal radiation is given as $I_{atm}(\lambda, T_{amb}) = \varepsilon_{atm}(\lambda, \theta) I_B(\lambda, T_{amb})$, where $\varepsilon_{atm}(\lambda, \theta)$ is the emissivity of the atmosphere and $I_B(\lambda, T_{amb})$ is the spectral emissivity of a black body. Therefore, the atmospheric radiation power absorbed by the surface can be calculated as:

$$P_{atm}(T_{amb}) = A_r \int_0^{\infty} \int_0^{2\pi} \int_0^{\frac{\pi}{2}} \alpha_r(\lambda, \theta) I_B(\lambda, T_{amb}) \, \varepsilon_{atm}(\lambda, \theta) \cos\theta \sin\theta \, d\theta d\phi d\lambda, \quad (3)$$

where $\alpha_r(\lambda, \theta)$ is the absorptivity of the surface, which can be replaced by its emissivity $\varepsilon_r(\lambda, \theta)$ according to Kirchhoff's law of thermal radiation (Supplementary Box1).

Some gas species in the atmosphere have dominant emissive characteristics over several wavelength bands. For instance, emission and absorption by H_2O are strong in the wavelengths at $\lambda \sim 6$ μm, and CO_2 has a strong emission band at $13 \leq \lambda \leq 16$ μm. Overall, several molecular emission bands give rise to atmospheric emissivity $\varepsilon_{atm}(\lambda, \theta)$, which is high over a broad wavelength range $0.3 \leq \lambda \leq 20$ μm except for some bands in the AW at $8 \leq \lambda \leq 13$ μm because very few electromagnetic waves are emitted by or absorbed by the atmosphere in this region (Fig. 1c). Coincidentally, the AW aligns with the peak wavelength (~9 μm) of blackbody radiation at a typical terrestrial ambient temperature (~300 K). The wavelength region thus provides a channel for radiative cooling, through which thermal radiation can propagate to the universe without being absorbed by the atmosphere.

$\varepsilon_{atm}(\lambda, \theta)$ significantly influences the effectiveness of radiative coolers (Eq. 3). Therefore, factors that affect $\varepsilon_{atm}(\lambda, \theta)$, and methods to measure it, should be understood. θ is a key factor that affects $\varepsilon_{atm}(\lambda, \theta)$[4,5]. Basically, as θ increases, $\varepsilon_{atm}(\lambda, \theta)$ increases because the atmospheric transparency decreases as the horizontal direction is approached. Slight changes in θ result in negligible differences in $\varepsilon_{atm}(\lambda, \theta)$ at small θ, but significant differences at large θ; for example, angle-dependent emissivity $\varepsilon_{atm}(\lambda, \theta)$ can be estimated from a given atmospheric emissivity $\varepsilon_{atm}(\lambda, 0)$ in the zenith direction as[4]:

$$\varepsilon_{atm}(\lambda, \theta) = 1 - \left(1 - \varepsilon_{atm}(\lambda, 0)\right)^{1/cos\theta} . \quad (4)$$

$\varepsilon_{atm}(\lambda, \theta)$ also depends on other factors, including geographical location, climate, and weather conditions. Absorption by H_2O vapor is dominant in the AW, so $\varepsilon_{atm}(\lambda, \theta)$ is quite sensitive to atmospheric humidity (Fig. 1c). Therefore, many studies have quantified correlations of between $\varepsilon_{atm}(\lambda, \theta)$, H_2O vapor pressure and ambient temperature in different regional and seasonal conditions[6–8]. Practically, $\varepsilon_{atm}(\lambda, \theta)$ can also be obtained from experimental measurements[7,9] or by using computer code[10,11] that were developed from models that account for different climates and regions.

Insolation

During the night, sub-ambient radiative cooling can be easily obtained[5,12,13]. However, during the day, absorption of sunlight by surfaces heats them and significantly counteracts radiative cooling [3,14]. Above the atmosphere, sunlight has a spectral distribution that is close to that of an ideal blackbody that has a surface temperature of 5778 K (Fig. 1b). However, while travelling through the atmosphere to the ground, the solar spectrum is unevenly attenuated by scattering and absorption, so the spectral solar irradiance $I_{solar}(\lambda, T)$ is very different from the blackbody spectrum[2]. $I_{solar}(\lambda, T)$ that considers the relative optical path length through the atmosphere can be calculated using models that consider the air mass (AM) coefficient. In particular, AM 1.5, which corresponds to $\theta = 48.2°$, is widely used to represent the average atmosphere at mid-latitudes. Thus, the solar irradiance power P_{solar} absorbed by the surface can be calculated as

$$P_{solar} = A_r \cos\theta \int_0^{\infty} \alpha_r(\lambda, \theta) I_{solar}(\lambda, T)\, d\lambda, \quad (5)$$

where $0 \leq \alpha_r(\lambda,\theta) \leq 1$ is the absorbance. In general, the absorbed solar power has the most significant effect on the daytime radiative cooling effect. Insolation at AM 1.5 can reach up to 1,000 $W{\cdot}m^{-2}$, so if the radiative cooler absorbs only 10% of it, the additional heat flux of 100 $W{\cdot}m^{-2}$ may cancel out the overall cooling effect. Therefore, daytime radiative coolers must have minimal absorption at $0.3 \leq \lambda \leq 2.5$ μm, where the insolation is concentrated.

Non-radiative heat transfer

Conduction and convection are important non-radiative heat-exchange mechanisms (Box 1). They result from the interaction between the radiative coolers and surrounding environment, such as the wind and the temperature difference between the radiative coolers and the ambient. The non-radiative heat transfer $P_{non-rad}$ can be mathematically expressed as

$$P_{non-rad} = A_r h (T_{amb} - T_s), \quad (6)$$

where h is the lumped heat-transfer coefficient that combines convection and conduction, and T_S is the surface temperature of the object to be cooled. $P_{non-rad}$ can be beneficial for cooling applications if $T_S > T_{amb}$, as in solar cells[15] and power plants[16]. However, if $T_S < T_{amb}$, then $P_{non-rad}$ acts counter to cooling. Methods to reduce this effect include using convection shields[17] and thermal insulation[18].

h can be determined empirically to estimate the loss to $P_{non-rad}$. For example, the effect of

wind speed V_{wind} on heat transfer coefficients at the outer surface can be expressed as a linear correlation[19–21]

$$h = a + bV_{wind}, \tag{6}$$

where the fitting parameters of a and b are obtained experimentally[22] or theoretically[23].

Ideal radiative cooling: broadband and selective emitter

Knowledge of the four heat-exchange processes that determine the net cooling effect of the daytime radiative cooler has been exploited to design two types of ideal radiative coolers for the maximum radiative cooling effect: a *broadband emitter* that emits in all of the electromagnetic spectrum except at wavelengths emitted by the sun, and a *selective emitter* that emits electromagnetic waves only in AW. The two types of radiative cooler provide ideal radiative cooling under different temperature conditions.

Both radiative coolers provide the ideal radiative cooling under different temperature conditions. The broadband emitter is preferred in above-ambient situations, because it emits a significant amount of energy to outer space. However, this type of device can also absorb the spectral emission from surroundings according to Kirchhoff's law, and this process can be impede in sub-ambient cooling. Consequently, the net cooling potential of the broadband emitter drastically decreases as the ambient temperature decreases (Supplementary Figure 1d).

In contrast, a selective emitter could exhibit more cooling potential than a broadband emitter in sub-ambient conditions, because it emits selectively to minimize the incoming radiative flux (Supplementary Figure 1d). Therefore, maximum radiative cooling effect for different purposes and environmental conditions, requires choice of materials and structures that have appropriate ideal radiative cooling curves.

Supplementary Note 2. Heat transfer

Blackbody radiation

A blackbody is an ideal object that absorbs or emits all incident radiation irrespective of wavelength or incident angle. The spectral intensity of blackbody is given by Planck's law:

$$I_B(\lambda, T) = \frac{2hc_0^2}{\lambda^5\left[\exp\left(\frac{hc_0}{\lambda k_B T}\right)-1\right]}, \qquad \text{(B1)}$$

where T is the absolute temperature (K) of the blackbody, h is the Planck constant, k_B is the Boltzmann constant, and c_0 is the speed of light in vacuum. By integrating the spectral intensity over the whole wavelength range, the total emitted power E of the blackbody is described by the Stefan-Boltzmann law $E(T) = \sigma T^4$, where σ is the Stefan-Boltzmann constant.

Every physical object emits electromagnetic radiation. However, no physical surface can emit or absorb more energy than a blackbody does (Eq. B1) at the same temperature and wavelength. A blackbody is generally designated as a reference in describing emission of a physical surface. Therefore, we define two physical properties that characterize thermal emission of any object: the angular spectral emissivity $\varepsilon(\lambda, \phi, \theta, T)$, and the angular spectral absorptivity $\alpha(\lambda, \phi, \theta, T)$, which are measured as the spectral radiation emitted or absorbed respectively by a surface at T and λ, as a ratio of the emission of absorption by a blackbody surface.

Kirchhoff's law of thermal radiation

In thermodynamic equilibrium, where no net radiation heat transfer occurs, the emissivity of the any radiating body is equal to the absorptivity at a given temperature, $\varepsilon(\lambda, \phi, \theta) = \alpha(\lambda, \phi, \theta)$. This is known as Kirchhoff's law of thermal radiation, which is valid if the Lorentz reciprocity holds.

Heat conduction and convection

Conduction is heat transfer through a medium in which temperature gradient exists, or between media that have different temperatures; i.e., heat energy is transferred from more-energetic to less-energetic particles by molecular motions and collisions inside the matter. Conductive heat transfer can be quantified by Fourier's law of heat conduction, where the heat flux density q_{cond} is given by $q_{cond} = -k\nabla T$, where k denotes the thermal conductivity, and ∇T is the temperature gradient. In general, k is larger in solids, in which molecules are closely packed, than in liquids and gases. Typically, the thermal conductivity of air is 0.03 $W \cdot m^{-1} \cdot K^{-1}$, water is 0.61 $W \cdot m^{-1} \cdot K^{-1}$, and Copper is 384 $W \cdot m^{-1} \cdot K^{-1}$.

Convection is heat transfer by a moving fluid. The driving force of convection heat transfer is the superposition of random molecular motion and the motion of the bulk fluid. Convection is classified into two types depending on the phenomena that drive the motion of flow: *natural convection*, which is caused by internal forces; and *forced convection*, which is caused by internal and external sources. The heat flux density q_{conv} for convection is given by Newton's law of cooling: $q_{cond} = h(T_f - T)$, where h denotes the convection heat transfer coefficient, and T_f is the fluid temperature. h depends on various conditions, including surface geometry, the thermophysical properties of the fluid, and the fluid velocity. Typical ranges are $10 \leq h$

≤ 100 W·m^{-2}·K^{-1} in air and $500 \leq h \leq 10{,}000$ W·m^{-2}·K^{-1} in water.

References

1. Lord, S.D. 1992, NASA Technical Memor. 103957 - Google Scholar. https://scholar.google.com/scholar?hl=en&as_sdt=0%2C5&q=Lord%2C+S.D.+1992%2C+NASA+Technical+Memor.+103957&btnG=.

2. T. L Bergman, Theodore L. Bergman, Frank P. Incropera, David P. DeWitt, A. S. L. *Fundamentals of Heat and Mass Transfer*. (John Wiley & Sons, Ltd, 2011).

3. Raman, A. P., Anoma, M. A., Zhu, L., Rephaeli, E. & Fan, S. Passive radiative cooling below ambient air temperature under direct sunlight. *Nature* **515**, 540–544 (2014).

4. Granqvist, C. G. & Hjortsberg, A. Radiative cooling to low temperatures: General considerations and application to selectively emitting SiO films. *J. Appl. Phys.* **52**, 4205–4220 (1981).

5. Berdahl, P., Martin, M. & Sakkal, F. Thermal performance of radiative cooling panels. *Int. J. Heat Mass Transf.* **26**, 871–880 (1983).

6. Bliss, R. W. Atmospheric radiation near the surface of the ground: A summary for engineers. *Sol. Energy* **5**, 103–120 (1961).

7. Berdahl, P. & Fromberg, R. The thermal radiance of clear skies. *Sol. Energy* **29**, 299–314 (1982).

8. Sicart, J. E., Hock, R., Ribstein, P. & Chazarin, J. P. Sky longwave radiation on tropical Andean glaciers: Parameterization and sensitivity to atmospheric variables. *J. Glaciol.* **56**, 854–860 (2010).

9. Deering, D. W. & Leone, P. A sphere-scanning radiometer for rapid directional measurements of sky and ground radiance. *Remote Sens. Environ.* **19**, 1–24 (1986).

10. Selby, J. E. A. & McClatchey, R. A. Atmospheric Transmittance From 0 . 25 t 28 . 5pMm : Computer Code LOWTRAN 3. *Environ. Res. Pap.* **513**, 1–110 (1975).

11. Berk, A. *et al.* MODTRAN5: 2006 update. in *Algorithms and Technologies for Multispectral, Hyperspectral, and Ultraspectral Imagery XII* vol. 6233 62331F (2006).

12. Catalanotti, S. *et al.* The radiative cooling of selective surfaces. *Sol. Energy* **17**, 83–89 (1975).

13. Bartoli, B. *et al.* Nocturnal and diurnal performances of selective radiators. *Appl. Energy* **3**, 267–286 (1977).

14. Rephaeli, E., Raman, A. & Fan, S. Ultrabroadband photonic structures to achieve high-performance daytime radiative cooling. *Nano Lett.* **13**, 1457–1461 (2013).

15. Zhu, L., Raman, A., Wang, K. X., Anoma, M. A. & Fan, S. Radiative cooling of solar cells. *Optica* **1**, 32 (2014).

16. Sabbagh, J. A., Khalifa, A. M. A. & Olwi, I. A. Development of passive dry cooling system for power plants in arid land. *Sol. Energy* **51**, 431–447 (1993).

17. Nilsson, N. A., Eriksson, T. S. & Granqvist, C. G. Infrared-transparent convection shields for radiative cooling: Initial results on corrugated polyethylene foils. *Sol. Energy Mater.* **12**, 327–333 (1985).

18. Hu, M., Zhao, B., Li, J., Wang, Y. & Pei, G. Preliminary thermal analysis of a combined photovoltaic–photothermic–nocturnal radiative cooling system. *Energy* **137**, 419–430 (2017).

19. Chow, T. T. Performance analysis of photovoltaic-thermal collector by explicit dynamic model. *Sol. Energy* **75**, 143–152 (2003).

20. Sartori, E. Convection coefficient equations for forced air flow over flat surfaces. *Sol. Energy* **80**, 1063–1071 (2006).

21. Kumar, S. & Mullick, S. C. Wind heat transfer coefficient in solar collectors in outdoor conditions. *Sol. Energy* **84**, 956–963 (2010).

22. Golaka, A. & Exell, R. H. B. An investigation into the use of a wind shield to reduce the convective heat flux to a nocturnal radiative cooling surface. *Renew. Energy* **32**, 593–608 (2007).

23. Armstrong, S. & Hurley, W. G. A thermal model for photovoltaic panels under varying atmospheric conditions. *Appl. Therm. Eng.* **30**, 1488–1495 (2010).
